# Determinants of myocardial function characterized by CMR-derived strain parameters in left ventricular non-compaction cardiomyopathy

**DOI:** 10.1038/s41598-019-52161-1

**Published:** 2019-11-04

**Authors:** Mareike Gastl, Alexander Gotschy, Malgorzata Polacin, Valery Vishnevskiy, Dominik Meyer, Justyna Sokolska, Felix C. Tanner, Hatem Alkadhi, Sebastian Kozerke, Robert Manka

**Affiliations:** 1 Department of Cardiology, University Heart Center, University Hospital Zurich, Raemistrasse 100, 8091 Zurich, Switzerland; 2 Institute for Biomedical Engineering, University and ETH Zurich, Gloriastrasse 35, 8092 Zurich, Switzerland; 3 Department of Cardiology, Pulmonology and Vascular Medicine, Heinrich Heine University, Düsseldorf, Germany; 4 Institute of Diagnostic and Interventional Radiology, University Hospital Zurich, Raemistrasse 100, 8091 Zurich, Switzerland; 5 Department of Heart Diseases, Wroclaw Medical University, Wroclaw, Poland

**Keywords:** Heart failure, Cardiomyopathies

## Abstract

Clinical presentation of left ventricular non-compaction cardiomyopathy (LVNC) can be heterogeneous from asymptomatic expression to congestive heart failure. Deformation indices assessed by cardiovascular magnetic resonance (CMR) can determine subclinical alterations of myocardial function and have been reported to be more sensitive to functional changes than ejection fraction. The objective of the present study was to investigate the determinants of myocardial deformation indices in patients with LVNC. Twenty patients with LVNC (44.7 ± 14.0 years) and twenty age- and gender-matched controls (49.1 ± 12.4 years) underwent functional CMR imaging using an ECG-triggered steady state-free-precession sequence (SSFP). Deformation indices derived with a feature tracking algorithm were calculated including end-systolic global longitudinal strain (GLS), circumferential strain (GCS), longitudinal and circumferential strain rate (SR_ll_ and SR_cc_). Twist and rotation were determined using an in-house developed post-processing pipeline. Global deformation indices (GLS, GCS, SR_ll_ and SR_cc_) were significantly lower in patients with LVNC compared to healthy controls (all, p < 0.01), especially for midventricular and apical regions. Apical rotation and twist were impaired for LVNC (p = 0.007 and p = 0.012), but basal rotation was preserved. Deformation indices of strain, strain rate and twist correlated well with parameters of the non-compacted myocardium, but not with the total myocardial mass or the thinning of the compacted myocardium, e.g. r = 0.595 between GLS and the non-compacted mass (p < 0.001). In conclusion, CMR deformation indices are reduced in patients with LVNC especially in affected midventricular and apical slices. The impairment of all strain and twist parameters correlates well with the extent of non-compacted myocardium.

## Introduction

Left ventricular non-compaction cardiomyopathy (LVNC) is a congenital cardiomyopathy that arises from a premature arrest of myocardial compaction during embryogenesis^1^. As a result, it is phenotypically characterized by a thin, compacted epicardial layer in contrast to a prominent non-compacted endocardial layer with multiple trabeculations and deep intertrabecular recesses. Diagnosis of LVNC is usually made using transthoracic echocardiography (TTE) as main diagnostic tool by describing the compacted thin epicardial layer and a thicker-non-compacted endocardial layer with a ratio between non-compacted to compacted myocardium >2 in end-systole of a short-axis slice, a predominant localization in midventricular and apical segments and perfused intertrabecular recesses^2^. Cardiovascular magnetic resonance (CMR) is frequently used to confirm or rule out the diagnosis.

As clinical manifestation of LVNC can be heterogeneous potentially leading to chronic heart failure or ventricular arrhythmias, reliable follow-up strategies are warranted^3^. While left ventricular ejection fraction (LVEF) represents one parameter to assess myocardial function, there is growing evidence that the assessment of myocardial deformation indices (strain parameters) provides additional information in the clinical setting.

Strain parameters as assessed by speckle tracking echocardiography have already shown reduced values for patients with LVNC even when LVEF was preserved^4–6^. This indicates that strain parameters are more sensitive to functional changes of the heart. As CMR deformation analysis using feature tracking algorithm can be derived from standard, cine steady-state-free precession (SSFP) images without the need for additional, time-consuming sequences and due to its reproducible, in-plane image acquisition this approach may have potential advantages. From hypertrophic cardiomyopathy it is known that CMR strain indices can already be reduced even when LVEF is normal or supernormal and that they may also yield prognostic information for patients with dilated cardiomyopathy or myocardial infarction^7–9^.

As a recent study indicated that CMR-based GLS, GCS and GRS were impaired in LVNC patients^10^, the aim of the present study was to investigate in addition also strain rate and twist in LVNC patients as well as regional strain parameters and to determine their correlation with morphological aspects of LVNC.

## Material and Methods

The study was conducted in accordance to the Declaration of Helsinki and its later amendments. The study design was approved by the Ethics Committee of the Canton of Zurich and written informed consent was obtained. All data used for this study were handled anonymously.

### Study population

In total, 20 patients with LVNC were prospectively included into this study from our outpatient clinic, between October 2011 and July 2016. Before the acquisition of the CMR, the enrolled patients had been diagnosed with LVNC according to current recommendations for transthoracic echocardiography (TTE)^2,11^. TTE parameters defined the non-compacted to compacted ratio > 2 in systole in a short-axis view predominantly localized in midventricular and apical segments with additional color Doppler evidence of perfused intertrabecular recesses as well as absence of coexisting cardiac abnormalities^2^. CMR diagnostic criteria defined the ratio of the thickness of LV non-compacted myocardium to compacted myocardium as greater than 2.3 in any long-axis view during diastole^11^. The LVNC group as well as 20 additional age- and gender-matched healthy controls received CMR.

### Cardiovascular magnetic resonance

Imaging was conducted on a 1.5 T MR imaging system (Achieva, Philips Healthcare, Best, The Netherlands) using a 5-channel phased array coil. After scout and reference scans, functional and geometric assessment was achieved by electrocardiogram-triggered, cine steady-state-free precession (SSFP) images in standard long-axis geometries (two-, three- and four-chamber view) as well as in short-axis orientation with full ventricle coverage from basis to apex (repetition time (TR)/echo time (TE) = 3.3/1.6 ms, flip angle (FA) = 60°, spatial resolution = 8 × 1.5 × 1.5 mm^3^, 2 slices per breath-hold, minimum phases: 25). Late gadolinium enhanced imaging 15 minutes after contrast agent (Gadovist, Bayer Healthcare, Berlin, Germany 0.2 mmol/kg) administration was performed in order to detect myocardial scarring or fibrosis. Therefore, a gradient-spoiled turbo fast-field-echo sequence with a non-selective 180° inversion pre-pulse was acquired at end-diastole with anatomical reference taken from SSFP images.

### Post processing

CMR analyses were performed using GTVolume (GyroTools LLC, Zurich, Switzerland). Standard analysis of functional and geometric LV indices was obtained including left and right ventricular end-diastolic volume (LVEDV/RVEDV), left and right ventricular ejection fraction (LV-/RV-EF), indexed left ventricular compacted mass with papillary muscles (LVMi-C), indexed left ventricular compacted and non-compacted mass (LVMi-MM), indexed left ventricular non-compacted mass (LVMi-NC) as well as their ratio (LVMi-NC/MM)^12^. In addition, the end-diastolic extent of compacted (LV-C) and non-compacted (LV-NC) myocardium as well as their ratio were measured in one long axis geometry (LV-NC/C)^11^.

**Figure 1:**
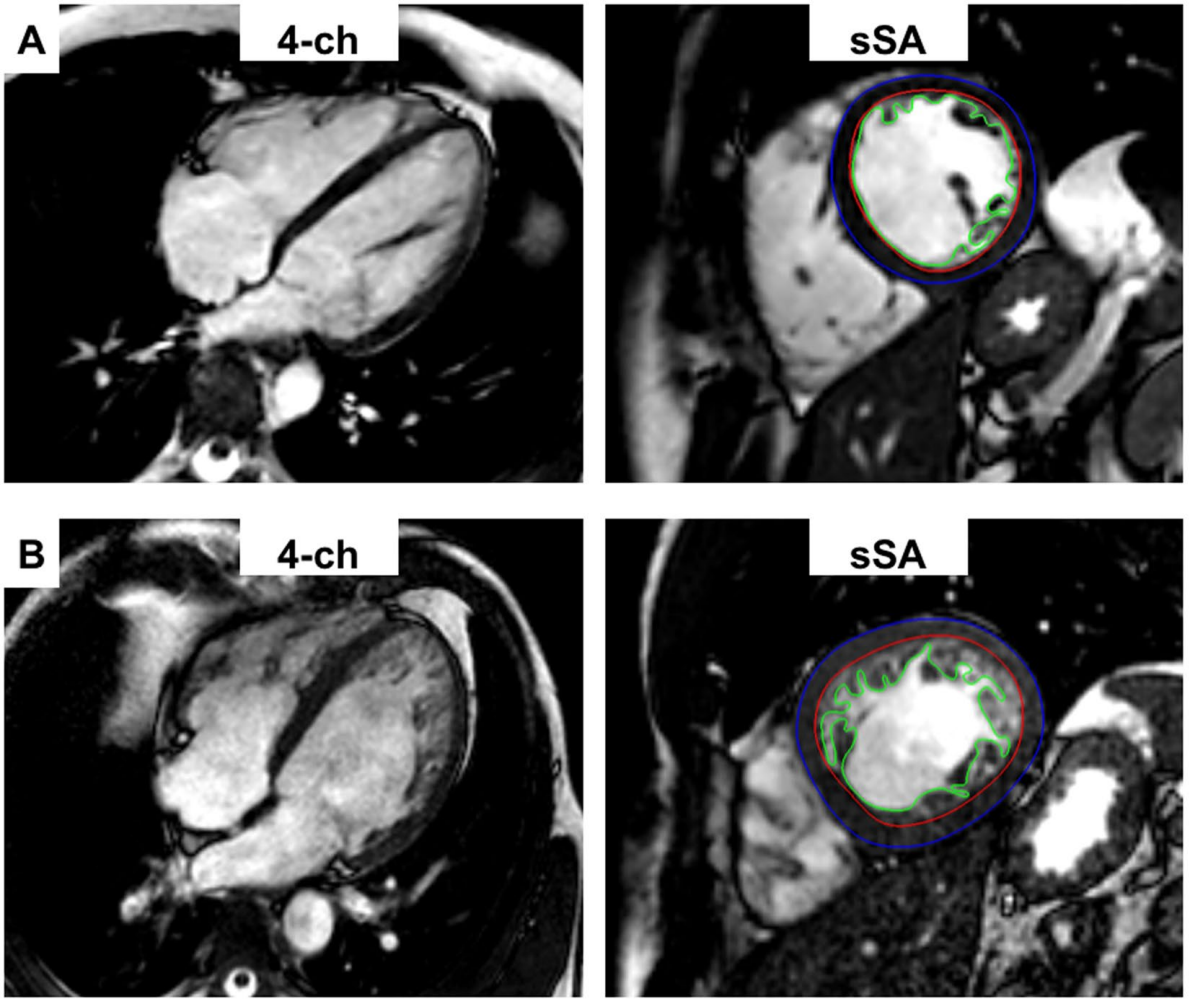
Segmentation of endocardial (red) and epicardial (blue) boarders as well as the non-compacted mass (green) for the calculation of masses and function in a control (**A**) and in a patient with LVNC (**B**). *ch*, chamber; *LVNC*, left ventricular non-compaction; *sSA*, single short axis.

Myocardial feature tracking (FT) analysis was performed using TomTec software (Image-Arena VA Version 3.0 and 2D Cardiac Performance Analysis MR Version 1.1.0; TomTec Imaging Systems, Unterschleissheim, Germany) on the basis of a previously established algorithm^13^. Cine images in long- and short-axis view were used for the analyses of end-systolic global longitudinal (GLS) and global circumferential (GCS) strain of the left ventricle, a global right ventricular longitudinal strain (RV-GLS) and longitudinal (SR_ll_) as well as circumferential (SR_cc_) peak diastolic strain rate. For strain analysis, endocardial contours were manually drawn independent of the cardiac cycle followed by subsequent software-driven automatic tracking of image features, such as tissue patterns or signal inhomogeneities (Fig. 1). Quality adjustment was performed afterwards and contours were amended manually if necessary. The phase of end-systolic strain was defined by aortic valve closure in three-chamber view for the long-axis geometries and the peak contraction around the nadir of the global strain curve for short-axis geometries^5^. In addition, intra- and interobserver agreement (both observers > 3 years’ experience in cardiac imaging) was achieved by repeating strain analysis on 28 randomly chosen subjects (14 LVNC and 14 controls).

In addition to strain parameters, peak apical and basal rotation as well as the end-systolic twist was obtained. End-systolic twist was defined by the subtraction of minimum basal rotation, obtained around end-systole, from maximum apical rotation around end-systole^14^.

### Customized motion tracking

As a result of unsatisfactory low reproducibility of the rotation parameters determined with the commercially available software, a custom-built, post-processing pipeline was built in-house to aim for more reproducible rotation parameters from short-axis cines images.

Dense motion fields via image registration were estimated using a previously established algorithm publicly available as Matlab toolbox^15,16^. The algorithm does not require myocardial segmentation and receives as an input N cine images that correspond to the different cardiac phases. The regularized registration problem is solved by estimating N spatial image transformations that minimize the nuclear norm of image patches. Such group-wise image similarity metric allows to non-parametrically enforce image alignment via patch correlation^17^. For the current short-axis images, cubic image interpolation was used, linear splines for displacement parametrization with grid stride of 4 pixels, L1-regularization of second order displacement derivatives in temporal domain with the weight of 5·10^–3^, vectorial total variation regularization in the spatial domain with weight of 5·10^–2^. Estimated displacement fields were then used to deform myocardial contour between different cardiac phases, e.g. displacement fields that map phase *t* to phase 0 were used to propagate the contour at phase 0: *c*_*t*_ = *c*_0_ ° *τ*_*t*,0_, (e.g end-systole to end-diastole).

After segmenting the myocardium in a basal, midventricular and apical short-axis slice by applying epi- and endocardial contours, its length (for endo- and epicardium) *s*^*l*^, and mean in-plane width *s*^*w*^ was calculated. Circumferential (${S}_{t}^{c}$) strain at the cardiac phase *t* was then defined as previously described^18^:

$${S}_{t}^{c}=\frac{{s}_{t}^{l}-{s}_{0}^{l}}{{s}_{0}^{l}},$$

where ${s}_{0}^{l}$ is the contour length at initial end-diastolic phase. In addition, rotation was estimated for a basal and an apical slice. To estimate the amount of rotation between cardiac phases 0 and *t*, a singular value decomposition was used to fit a rigid body motion model to displacement vectors inside the myocardium and then extracted Euler angle from the orthogonal rotation matrix.

### Statistical analysis

Statistical analysis was performed using SPSS 24.0 (SPSS Inc., Chicago, IL, US). Unless otherwise stated, continuous variables are presented as mean ± standard deviation (SD). Normal distribution was tested using the Shapiro-Wilk test. Categorical variables are reported as percentage. Data between the two different groups were analyzed by 2-sided unpaired Student’s t-tests for normally distributed data and Mann-Whitney *U*-test for not normally distributed data. For post-hoc analyses, the LVNC patient group was divided into two subgroups with either preserved (LVEF ≥ 50%) or reduced (LVEF < 50%) systolic function.

χ^2^ test was used to examine significant differences between nominal classifications. For intra- and interobserver agreement, coefficients of variation (CoV) were calculated by dividing the SD of the differences by the mean. In addition, intraclass correlation coefficients (ICC) were assessed using a model of absolute agreement. There was excellent agreement when ICC > 0.74, good when ICC = 0.60–0.74, fair when ICC = 0.40–0.59, and poor when ICC < 0.4^19^.

Pearsons correlation was performed to calculate correlations between functional and geometric CMR parameters. Two-tailed p-values below 0.05 were considered statistically significant.

## Results

### Patient characteristics

20 LVNC patients (mean age 44.7 ± 14.0 years) were included into this study. The age- and sex-matched 20 healthy control subjects showed similar body surface area (BSA) characteristics. Further clinical baseline characteristics are summarized in Table 1. Only 4 patients presented with small LGE (1x anterior, 1x anterolateral and 2x septal) and 14 did not show LGE. One patient did not receive contrast agent and for 1 patient, LGE images were non-diagnostic.

**Table 1:** Demographic characteristics of the study cohort

	LVNC (n = 20)	Controls (n = 20)	p-value
Age (years)	44.7 ± 14.0	49.1 ± 12.4	0.253
Male (%)	11 (55)	13 (65)	0.518
BSA (m^2^)	1.8 ± 0.2	1.9 ± 0.2	0.068
**Comorbidities**			
Diabetes, n(%)	1 (5)	1 (5)	1.000
Hypertension, n(%)	3 (15)	0 (0)	0.072
Hypercholesterolemia, n(%)	0 (0)	0 (0)	1.000
Renal failure (CKD > II), n(%)	0 (0)	0 (0)	1.000
CAD, n(%)	0 (0)	0 (0)	1.000
Previous PCI, n(%)	0 (0)	0 (0)	1.000
Previous CABG n(%)	0 (0)	0 (0)	1.000
Previous Stroke, n(%)	1 (5)	0 (0)	0.311
NYHA III n(%)	2 (10)	0 (0)	0.147
Family history LVNC, n (%)	7 (35)	0 (0)	0.004

*BSA*, body surface area; *CABG*, coronary artery bypass graft; *CAD*, coronary artery disease; *CKD*, chronic kidney disease; *LVNC*, left-ventricular non-compaction; *NYHA*, New York Heart Association; *PCI*, percutaneous coronary intervention.

LVNC patients had a significantly lower LVEF in comparison to the controls (Table 2), but according to recent heart failure guidelines mean LVEF in the patient group was still preserved^20^. Eight LVNC patients showed a (mid-range) reduced LVEF with values < 50%. Despite no difference in LVMi-C, there were significantly higher values in non-compacted parameters as well as their ratios for patients with LVNC. No differences could be seen for RV functional and geometric indices.

**Table 2:** CMR characteristics of LVNC patients and their controls.

Left ventricle	LVNC	Controls	p-value
LV-EF [%]	51.6 ± 8.6	62.1 ± 4.2	< 0.001
LVEDV [ml]	181.1 ± 63.1	152.1 ± 19.9	0.081
LV-C [mm]	5.4 ± 1.0	6.2 ± 1.0	0.014
LV-NC [mm]	14.6 ± 5.4	8.1 ± 2.2	< 0.001
LV-NC/C	2.8 ± 1.5	1.4 ± 0.3	< 0.001
LVMi-C [g/m^2^]	55.8 ± 15.3	52.9 ± 11.3	0.779
LVMi-NC [g/m^2^]	39.8 ± 24.9	11.9 ± 3.8	< 0.001
LVMi-MM [g/m^2^]	95.6 ± 33.1	64.8 ± 13.5	< 0.001
LVMi-NC/MM	0.40 ± 0.10	0.18 ± 0.04	< 0.001
**Right ventricle**			
RV-EF	53.8 ± 13.1	57.2 ± 4.9	0.429
RVEDV	157.5 ± 40.5	156.4 ± 22.9	0.919
RV GLS	−21.8 ± 7.4	−20.5 ± 4.9	0.491
Free wall longitudinal	−24.4 ± 10.2	−22.3 ± 8.6	0.482
Septum longitudinal	−16.2 ± 6.1	−15.0 ± 4.2	0.738

*C*, compacted with papillary muscles; *GLS*, global longitudinal strain; *LV*, left ventricular; *LVEDV*, left ventricular end-diastolic volume; *LVEF*, left ventricular ejection fraction; *LVMi*, left ventricular indexed mass; *MM*, compacted plus non-compacted, *NC*, non-compacted; *RV*, right ventricular; *RVEDV*, right ventricular end-diastolic volume; *RVEF*, right ventricular function.

### CMR - strain

**Figure 2:**
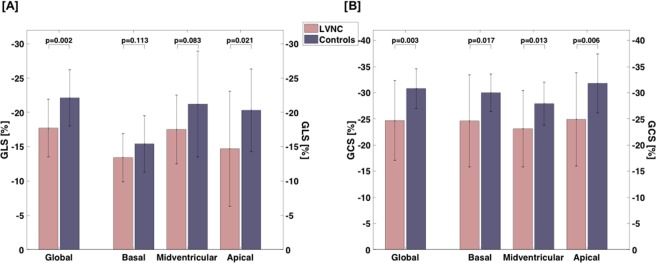
Differences of global and regional GLS [**A**] and GCS [**B**] between LVNC and controls. *GCS*, global circumferential strain; *GLS*, global longitudinal strain; *LVNC*, left ventricular non-compaction.

Feature tracking analysis revealed significantly lower GCS and GLS values for the LVNC group compared to controls (GLS LVNC vs. controls: −17.7 ± 4.2 vs. −22.1 ± 4.1%, p = 0.002; GCS LVNC vs. controls: −24.7 ± 7.6 vs. −30.8 ± 3.8, p = 0.003). On a regional level, GLS parameters were not different for basal and midventricular segments, but for apical ones (Fig. 2a). For GCS, significantly lower values were detected for basal, midventricular and apical slices with the highest differences for apical segments (Fig. 2b). ANOVA analysis indicated a difference of GLS between controls and LVNC patients with LVEF > 50% (N = 12) and LVEF < 50% (N = 8) (p = 0.002). In post-hoc Bonferroni correction, this difference only remained for the comparison of controls and the subgroup of patients with reduced LVEF < 50% (p = 0.001), but not between the two subgroups. For GCS, ANOVA indicated a difference between the groups as well (p < 0.001), which was most pronounced between controls and patients within the subgroup of reduced LVEF (p < 0.001), less pronounced between the two subgroups (p = 0.022). There was no difference between controls and patients within the subgroup of preserved LVEF > 50%. Three patients with LGE presented with GLS and GCS below the mean of the LVNC group.

Besides a correlation with LVEF, further correlation analyses revealed that GLS best correlated to parameters including the noncompacted areas (Table 3), such as LVMi-NC, LVMi-MM, LV-NC and LV-NC/C. The same applied for GCS. In addition, there was an association between decreasing thickness, but not mass, of compacted area (LV-C) and decreasing GCS (R = −0.449, p = 0.004).

**Table 3:** Correlation analysis between strain parameters (GLS, GCS) and left ventricular functional and dimensional parameters.

	GLS [%]		GCS [%]	
	R	p-value	R	p-value
LVEF [%]	−0.689	<0.001	−0.856	<0.001
LVEDV [ml]	0.567	<0.001	0.547	<0.001
LVMi-C [g/m^2^]	0.331	0.044	0.045	0.783
LVMi-NC [g/m^2^]	0.595	<0.001	0.57	<0.001
LVMi-MM [g/m^2^]	0.602	<0.001	0.457	0.003
LVMi-NC/MM	0.497	0.001	0.559	<0.001
LV-C [mm]	−0.312	0.050	−0.449	0.004
LV-NC [mm]	0.517	0.001	0.421	0.007
LV-NC/C	0.524	0.001	0.541	<0.001

*C*, compacted with papillary muscles; *GCS*, global circumferential strain; *GLS*, global longitudinal strain; *LV*, left ventricular; *LVEDV*, left ventricular end-diastolic volume; *LVEF*, left ventricular ejection fraction; *LVMi*, left ventricular indexed mass; *MM*, compacted plus non-compacted, *NC*, non-compacted.

Mean differences ±1.96 SD, CoV and ICC for GLS and GCS divided by control and diseased groups are summarized in Table 4 for intra- and interobserver variability. There was good to excellent inter- and intraobserver reproducibility for GLS and GCS in the healthy and diseased groups (ICC 0.68–0.97). Intraobserver agreement showed higher reproducibility than interobserver agreement.

**Table 4:** Intra- and interobserver reproducibility (N = 14 each) for the different deformation parameters divided by LVNC patients and healthy controls.

	Healthy							
	Intraobserver			Interobserver				
	Mean difference ± 1.96 SD	CoV [%]	ICC (95% CI)	Mean difference ± 1.96 SD	CoV [%]		ICC (95% CI)	
GLS [%]	−0.06 ± 2.86	6.6	0.97 (0.91–0.99)	−1.28 ± 4.32	9.6		0.9 (0.64–0.97)	
GCS [%]	2.32 ± 3.02	5.1	0.83 (−0.16–0.96)	3.38 ± 4.19	7.2		0.68 (−0.24–0.92)	
SR_ll_ [s^−1^]	−0.01 ± 0.19	8.2	0.99 (0.95–1.0)	0.00 ± 0.266	11.3		0.98 (0.92–0.99)	
SR_cc_ [s^−1^]	−0.07 ± 0.16	4.7	0.98 (0.89–0.97)	−0.15 ± 0.38	11.5		0.9 (0.54–0.97)	
Apical rotation [°]	−0.02 ± 0.60	8.4	0.99 (0.97–1.0)	−0.08 ± 0.82	11.5		0.98 (0.93–0.99)	
Basal rotation [°]	0.16 ± 0.54	12.8	0.99 (0.96–1.0)	0.21 ± 0.80	19.3		0.98 (0.92–0.99)	
**Diseased**								
	**Intraobserver**			**Interobserver**				
	**Mean difference ± 1.96 SD**	**CoV [%]**	**ICC (95% CI)**	**Mean difference ± 1.96 SD**		**CoV [%]**		**ICC (95% CI)**
GLS [%]	−0.06 ± 2.68	7.2	0.96 (0.87–0.99)	−1.64 ± 5.35		13.9		0.77 (0.26–0.93)
GCS [%]	1.75 ± 3.10	6.4	0.98 (0.85–1.0)	3.1 ± 3.69		7.8		0.95 (0.09–0.99)
SR_ll_ [s^−1^]	−0.02 ± 0.11	6.4	0.99 (0.97–1.0)	−0.03 ± 0.24		14.2		0.95 (0.83–0.98)
SR_cc_ [s^−1^]	−0.02 ± 0.13	5.3	0.99 (0.98–1.0)	−0.07 ± 0.13		5.4		0.99 (0.89–1.0)
Apical rotation [°]	−0.12 ± 0.98	20.2	0.95 (0.86–0.98)	−0.09 ± 1.04		21.3		0.95 (0.84–0.98)
Basal rotation [°]	−0.12 ± 0.42	13.1	0.99 (0.96–1.0)	−0.03 ± 0.37		11.8		0.99 (0.98–1.0)

*CoV*, coefficient of variation*; GCS*, global circumferential strain; *GLS*, global longitudinal strain; *ICC*, intraclass correlation coefficient; *LVNC*, left ventricular non-compaction; *SD*, standard deviation; *SRcc*, peak circumferential strain rate, *SR*_*ll*_, peak longitudinal strain rate.

As a second result of the newly-developed tool to analyze rotational parameters, GCS was calculated as well. There was a significant difference in global GCS for LVNC (LVNC vs. controls: −19.1 ± 6.4% vs. −25.2 ± 2.8, p < 0.001). Statistical significance of differences increased again from basal (−21.5 ± 8.1 vs. −27.1 ± 4.2, p = 0.017), to midventricular (18.5 ± 5.7 vs. 22.9 ± 4.0, p = 0.007) and apical GCS (−19.2 ± 7.5 vs. 27.5 ± 4.1, p < 0.001). In addition, there was excellent intraobserver reproducibility for the healthy (CoV 4.1%, ICC = 0.87) and diseased group (CoV 6.2%, ICC = 0.93). Interobserver reproducibility showed similar results (healthy: CoV 4.2%, ICC = 0.93; LVNC: CoV 6.8%, ICC = 0.98).

### CMR – strain rate

**Figure 3:**
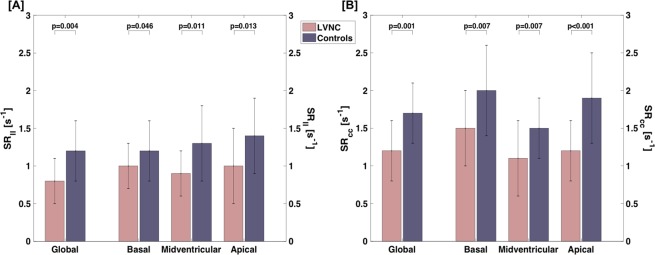
Differences of global and regional SR_ll_ [**A**] and SR_cc_ [**B**] between LVNC and controls. *LVNC*, left ventricular non-compaction; *SR*_*cc*_, peak-diastolic circumferential strain rate; *SR*_*ll*_, peak-diastolic longitudinal strain rate.

Like GLS and GCS, peak diastolic SR_ll_ and SR_cc_ were significantly lower for LVNC compared to controls (Fig. 3a,b). On a regional level, the difference was most pronounced in midventricular and apical slices for SR_ll_ and in all slices for SR_cc_ (Fig. 3a,b). ANOVA analysis indicated a difference of SR_ll_ between controls and both LVNC subgroups (p = 0.004). In post-hoc Bonferroni correction, this difference only remained for the comparison of controls and patients within the subgroup of reduced LVEF (p = 0.004), but not between the two subgroups. For SR_cc_, ANOVA indicated a difference between the groups as well (p < 0.001), which was most pronounced between controls and patients within the subgroup of reduced LVEF (p < 0.001), less pronounced between the two subgroups (p = 0.004). There was no difference between controls and patients within the subgroup of preserved LVEF. Like for strain, three patients with LGE presented with SR_ll_ and SR_cc_ below the mean of the LVNC group.

Correlation analyses revealed the best correlation of SR_ll_ and SR_cc_ to parameters of the non-compacted myocardium, such as LV-NC or LVMi-NC (Table 5). For both parameters, there was no correlation to the compacted myocardium.

**Table 5:** Correlation analysis between strain rate parameters (SR_ll_, SR_cc_) and left ventricular functional and dimensional parameters.

	SR_ll_ (s^−1^)		SR_cc_ (s^−1^)	
	R	p-value	R	p-value
LVEF [%]	0.546	<0.001	0.756	<0.001
LVEDV [ml]	−0.472	0.002	−0.486	0.001
LVMi-C [g/m^2^]	−0.281	0.079	−0.090	0.581
LVMi-NC [g/m^2^]	−0.467	0.002	−0.532	<0.001
LVMi-MM [g/m^2^]	−0.486	0.001	−0.449	0.004
LVMi-NC/MM	−0.395	0.012	−0.542	<0.001
LV-C [mm]	0.027	0.868	0.259	0.107
LV-NC [mm]	−0.491	0.001	−0.473	0.002
LV-NC/C	−0.406	0.009	−0.469	0.002

*C*, compacted with papillary muscles; *LV*, left ventricular; *LVEDV*, left ventricular end-diastolic volume; *LVEF*, left ventricular ejection fraction; *LVMi*, left ventricular indexed mass; *MM*, compacted plus non-compacted; *NC*, non-compacted; *SRcc*, peak circumferential strain rate, *SR*_*ll*_, peak longitudinal strain rate.

Mean differences ±1.96 SD, CoV and ICC for SR_ll_ and SR_cc_ divided by healthy and diseased groups are summarized in Table 4 for intra- and interobserver variability. There was good to excellent reproducibility for these parameters in the healthy and diseased groups (ICC 0.9–0.99). Like for global strain, intraobserver agreement showed higher reproducibility than interobserver agreement.

### CMR – rotation

Using the commercially available strain analysis software, CoV for basal rotation was 83.0 (ICC = 0.51) and 101.9% (ICC = 0.65) for intra- and interobserver agreement for the healthy group and 98.6 (ICC = 0.35) and 98.0% (ICC = 0.34) for the diseased group. Results for the apical rotation showed a CoV of 65.2% (ICC = 0.68) and 72.2% (ICC = 0.77) for the healthy, and 51.7 (ICC = 0.55) as well as 46.7% (ICC = 0.68) for the diseased group.

The in-house written MATLAB-based script showed excellent reproducibility for rotational parameters in the healthy and diseased groups (ICC 0.95–0.99) (Table 4). Reproducibility was higher in apical segments for the healthy and in basal segments for the diseased group.

**Figure 4:**
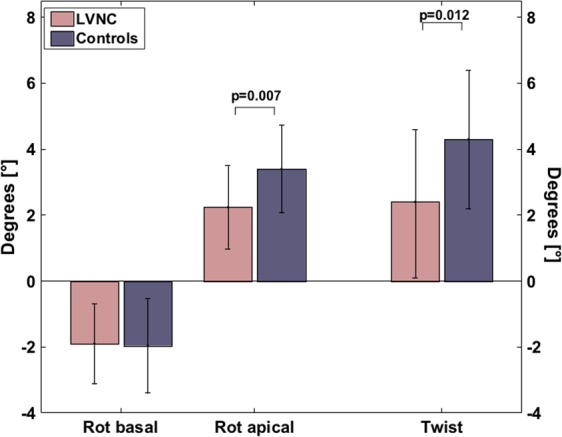
Basal and apical rotation as well as end-systolic twist between LVNC and controls.*LVNC*, left ventricular non-compaction; *rot*, rotation.

Analyses showed that there was no difference in basal rotation, but a significant difference in maximum apical rotation with lower values for the LVNC group (p = 0.007) (Fig. 4). This difference remained for end-systolic twist with lower values for the LVNC group (p = 0.012). ANOVA analysis indicated a difference of apical rotation and twist between controls and both LVNC subgroups (p = 0.005 and p = 0.007). In post-hoc Bonferroni correction, this difference only remained for the comparison of controls and patients within the subgroup of reduced LVEF < 50% for apical rotation (p = 0.004) and twist (p = 0.006). The 4 patients with LGE showed heterogeneous values in rotational analyses with values below or above the mean.

Similar to strain values, twist correlated well with parameters of the non-compacted myocardium, e.g. LV-NC/C (r = −0.454, p = 0.003) (Table 6).

**Table 6:** Correlation analysis between twist and left ventricular functional and dimensional parameters.

	Twist [%]	
	R	p-value
LVEF [%]	0.615	<0.001
LVEDV [ml]	−0.247	0.125
LVMi-C [g/m^2^]	0.018	0.914
LVMi-NC [g/m^2^]	−0.407	0.009
LVMi-MM [g/m^2^]	−0.304	0.057
LVMi-NC/MM	−0.443	0.004
LV-C [mm]	0.213	0.187
LV-NC [mm]	−0.402	0.010
LV-NC/C	−0.454	0.003

*C*, compacted with papillary muscles; *LV*, left ventricular; *LVEDV*, left ventricular end-diastolic volume; *LVEF*, left ventricular ejection fraction; *LVMi*, left ventricular indexed mass; *MM*, compacted plus non-compacted; *NC*, non-compacted.

## Discussion

The present study provides CMR myocardial deformation indices in patients with LVNC compared to controls on a global and regional level. GLS and GCS, as well as SR_ll_ and SR_cc_ were found to be significantly impaired in LVNC, especially in midventricular and apical segments with a good correlation to parameters of the non-compacted myocardium. By implementing a software for assessing ventricular rotation, impaired apical rotation and twist with good reproducibility could be shown as well.

During its heterogeneous course, LVNC may lead to heart failure or arrhythmic events^21,22^. Besides TTE, CMR is frequently used to confirm the diagnosis of LVNC or for follow-up strategies. As additional parameter to LVEF, CMR-derived strain can easily be assessed by post-processing standard cine images without the need for further sequences like tagging, or displacement-encoding (DENSE). In addition, CMR yields the advantage of a reproducible, in-plane image acquisition. Therefore, these parameters can be of additional value for the description of functional changes in LVNC^7,23^.

Our results are similar to those obtained by speckle tracking echocardiography that showed impaired GLS, GCS and strain rate parameters in patients with LVNC^6,14^. In addition, a global strain reduction has already been shown in a CMR study of children and adolescents with LVNC^6,10^. However, the disease is mainly manifested in apical and midventricular regions which is mirrored in regional CMR-derived strain parameters for the first time. This can be underlined by the new correlation of strain parameters to indices of noncompacted masses^2^. Therefore, GLS is preserved in basal regions (Fig. 2) and the difference to controls increases from basal to apical regions^6^. Surprisingly, GCS, SR_ll_ and SR_cc_ showed already reduced values in basal regions, although the difference to controls from basal to apical regions increases as well. One influencing factor of this finding can be the heterogeneous group of LVNC patients with different LVEF. Indeed, dividing the group of LVNC patients according to LVEF, patients within the subgroup of reduced LVEF < 50% showed the most prominent reduction in all strain parameters. From hypertrophic cardiomyopathy it is known that CMR strain indices can already be reduced even when LVEF is normal or supernormal, which leads to the hypothesis that strain indices are more sensitive to capture the 3-dimensional nature of myocardial function in certain clinical settings^7^. In the present cohort, strain parameters of LVNC patients within the subgroup of preserved LVEF were not statistically different from strain parameters of controls after Bonferroni correction. However, their values were below values for normal controls and especially GLS and SR_ll_ were not different to patients within the subgroup of reduced LVEF thereby indicating a functional state between healthy and diseased LVNC patients with impaired LVEF.

For a reliable application in clinical settings, reproducibility is important. Overall strain and strain rate assessment showed reproducible assessment with good to excellent consistency of measurements (ICC between 0.68 and 0.99), which is comparable to literature^24^. In addition, the newly-developed tool for rotational analyses was able to provide GCS results with good reproducibility. Although mean differences were slightly higher for GCS, the tendency of over- or underestimating the parameters pointed in the same direction for healthy and diseased patients with GLS being lower for repeated measurements and GCS being higher therefore preserving differences of parameters between the groups. Surprisingly and in contrast to existing literature, parameters of rotation were not reproducible using standard feature tracking software^19^. This might be due to the fact that an average of three measurements was used to calculate a global torsion value in previous studies about reproducibility of torsion. In addition, the current study chose to report inter-observer agreement as well^19^. Nevertheless, using the customized pipeline for the assessment of rotational parameters, similar CoV and ICC could be obtained as presented in literature with the advantage of only one segmentation.

As already shown using only speckle-tracking echocardiography so far, apical rotation and twist was impaired in LVNC^6,14,25^. Due to the still preserved basal rotation, a previously described ‘solid body rotation’ with near absent LV twist could not be confirmed in the present study^14^. This may be influenced by the different image planning of TTE and CMR. Reproducibility was poorest for diseased patients in apical rotation. Given that the disease mainly affects midventricular and apical regions with lower values, the higher CoV is not surprising^2^.

### Limitations

As the incidence of LVNC in the normal population is low, a limitation of the current work is the relatively small sample size of patients with LVNC, especially for the division according to an LVEF threshold^11^. However, this limited population already showed promising data for the description of myocardial strain indices in patients with LVNC and was comparable to patient numbers previously published in the literature^6,14^.

As an inherent limitation, FT algorithms can only distinguish the endo- or epicardial interfaces of the myocardium and are, therefore, unable to assess transmural variations of the myocardial strain^18^. In contrast, echocardiographic speckle tracking based assessment of layered strain has already shown different subendo- and subepicardial dynamics in patients after aortic valve replacement^26^. Since LVNC is considered to have differential impact on the myocardial layers, an investigation of the transmural variation of strain would be of interest for future studies.

In addition, global radial and segmental strain were not obtained due to recent evidence of their lower reproducibility^24^.

## Conclusion

CMR deformation indices including GLS, GCS and strain rate parameters were reduced in patients with LVNC especially in affected midventricular and apical slices and correlated well with parameters of the non-compacted myocardium. By using a novel approach for post-processing CMR data, reduced twist and apical rotation with a good reproducibility could be shown. CMR-derived deformation indices may show added value to assess functional impairment in LVNC, even when LVEF is preserved.

## Data Availability

The datasets generated during and/or analysed during the current study are available from the corresponding author on reasonable request.

## References

[1] Chin TK, Perloff JK, Williams RG, Jue K, Mohrmann R (1990). Isolated noncompaction of left ventricular myocardium. A study of eight cases. Circulation.

[2] Jenni R, Oechslin E, Schneider J, Attenhofer Jost C, Kaufmann PA (2001). Echocardiographic and pathoanatomical characteristics of isolated left ventricular non-compaction: a step towards classification as a distinct cardiomyopathy. Heart.

[3] Ritter M (1997). Isolated noncompaction of the myocardium in adults. Mayo Clin. Proc..

[4] Kalam K, Otahal P, Marwick TH (2014). Prognostic implications of global LV dysfunction: a systematic review and meta-analysis of global longitudinal strain and ejection fraction. Heart.

[5] Voigt J-U (2015). Definitions for a common standard for 2D speckle tracking echocardiography: consensus document of the EACVI/ASE/Industry Task Force to standardize deformation imaging. Eur. Hear. J. - Cardiovasc. Imaging.

[6] Bellavia D (2010). Speckle myocardial imaging modalities for early detection of myocardial impairment in isolated left ventricular non-compaction. Heart.

[7] Smiseth OA, Torp H, Opdahl A, Haugaa KH, Urheim S (2016). Myocardial strain imaging: how useful is it in clinical decision making?. Eur. Heart J..

[8] Eitel I (2018). Cardiac Magnetic Resonance Myocardial Feature Tracking for Optimized Prediction of Cardiovascular Events Following Myocardial Infarction. JACC Cardiovasc. Imaging.

[9] Buss SJ (2015). Assessment of myocardial deformation with cardiac magnetic resonance strain imaging improves risk stratification in patients with dilated cardiomyopathy. Eur. Hear. J. – Cardiovasc. Imaging.

[10] Nucifora G (2017). Cardiac magnetic resonance evaluation of left ventricular functional, morphological, and structural features in children and adolescents vs. young adults with isolated left ventricular non-compaction. Int. J. Cardiol..

[11] Petersen SE (2005). Left Ventricular Non-Compaction. J. Am. Coll. Cardiol..

[12] Jacquier A (2010). Measurement of trabeculated left ventricular mass using cardiac magnetic resonance imaging in the diagnosis of left ventricular non-compaction. Eur. Heart J..

[13] Hor KN (2010). Comparison of magnetic resonance feature tracking for strain calculation with harmonic phase imaging analysis. JACC. Cardiovasc. Imaging.

[14] van Dalen BM (2008). Left ventricular solid body rotation in non-compaction cardiomyopathy: A potential new objective and quantitative functional diagnostic criterion?. Eur. J. Heart Fail..

[15] Vishnevskiy V, Gass T, Szekely G, Tanner C, Goksel O (2017). Isotropic Total Variation Regularization of Displacements in Parametric Image Registration. IEEE Trans. Med. Imaging.

[16] Vishnevskiy, V. Deformable image registration (alignment) toolbox. *GitHub repository* (2018).

[17] Huizinga W (2016). PCA-based groupwise image registration for quantitative MRI. Med. Image Anal..

[18] Pedrizzetti G, Claus P, Kilner PJ, Nagel E (2016). Principles of cardiovascular magnetic resonance feature tracking and echocardiographic speckle tracking for informed clinical use. J. Cardiovasc. Magn. Reson..

[19] Kowallick JT (2016). Inter-study reproducibility of left ventricular torsion and torsion rate quantification using MR myocardial feature tracking. J. Magn. Reson. Imaging.

[20] Ponikowski P (2016). 2016 ESC Guidelines for the diagnosis and treatment of acute and chronic heart failure. Eur. J. Heart Fail..

[21] Stämpfli SF (2017). Prognostic power of NT-proBNP in left ventricular non-compaction cardiomyopathy. Int. J. Cardiol..

[22] Oechslin EN, Attenhofer Jost CH, Rojas JR, Kaufmann PA, Jenni R (2000). Long-term follow-up of 34 adults with isolated left ventricular noncompaction: a distinct cardiomyopathy with poor prognosis. J. Am. Coll. Cardiol..

[23] Andre F (2015). Age- and gender-related normal left ventricular deformation assessed by cardiovascular magnetic resonance feature tracking. J. Cardiovasc. Magn. Reson..

[24] Maceira AM (2018). Feasibility and reproducibility of feature-tracking-based strain and strain rate measures of the left ventricle in different diseases and genders. J. Magn. Reson. Imaging.

[25] Phillips AA, Cote AT, Bredin SS, Warburton DE (2012). Heart Disease and Left Ventricular Rotation – A Systematic Review and Quantitative Summary. BMC Cardiovasc. Disord..

[26] Fung MJ, Thomas L, Leung DY (2019). Alterations in Layer-Specific Left Ventricular Global Longitudinal and Circumferential Strain in Patients With Aortic Stenosis: A Comparison of Aortic Valve Replacement versus Conservative Management Over a 12-Month Period. J. Am. Soc. Echocardiogr..

